# Profile of treatment-related complications in women with clinical stage IB-IIB cervical cancer: A nationwide cohort study in Japan

**DOI:** 10.1371/journal.pone.0210125

**Published:** 2019-01-07

**Authors:** Hiroko Machida, Koji Matsuo, Akiko Furusawa, Tsunekazu Kita, Ryo Kitagawa, Mikio Mikami

**Affiliations:** 1 Department of Obstetrics and Gynecology, Tokai University School of Medicine, Kanagawa, Japan; 2 Division of Gynecologic Oncology, Department of Obstetrics and Gynecology, University of Southern California, Los Angeles, CA, United States of America; 3 Norris Comprehensive Cancer Center, University of Southern California, Los Angeles, CA, United States of America; 4 Department of Gynecology, Metropolitan Cancer and Infectious Diseases Center Komagome Hospital, Tokyo, Japan; 5 Department of Obstetrics and Gynecology, Nara Prefecture General Medical Center, Nara, Japan; 6 Department of Obstetrics and Gynecology, Tohoku Medical and Pharmaceutical University, Miyagi, Japan; University of South Alabama Mitchell Cancer Institute, UNITED STATES

## Abstract

**Objective:**

To examine clinico-pathological factors associated with surgical complications and postoperative therapy for clinical stage IB-IIB cervical cancer.

**Methods:**

This nationwide multicenter retrospective study examined women with clinical stage IB-IIB cervical cancer who underwent radical hysterectomy plus pelvic and/or para-aortic lymphadenectomy between 2008–2009 at 87 institutions of the Japanese Gynecologic Oncology Group (*n* = 693). Multivariate models were used to identify independent predictors of perioperative grade 3–4 complications and bladder dysfunction.

**Results:**

The overall intraoperative and postoperative complication rates were 3.3% and 9.8%, respectively. Clinical stage was not associated with perioperative complications (*P* = 0.15). Radiotherapy-based adjuvant therapy was significantly associated with an increased risk of postoperative complications (radiotherapy alone: adjusted-odds ratio [OR] 3.19, 95% confidence interval [CI] 1.46–6.99, *P* = 0.004; radiotherapy plus chemotherapy: adjusted-OR 3.26, 95%CI 1.66–6.41, *P* = 0.001), whereas chemotherapy was not (*P* = 0.45). Nerve-sparing surgery significantly reduced the risk of postoperative bladder dysfunction (adjusted-OR 0.57, 95%CI 0.37–0.90, *P* = 0.02) whereas adjuvant chemotherapy increased the risk of bladder dysfunction (adjusted-OR 2.06, 95%CI 1.16–3.67, *P* = 0.01). Among women receiving adjuvant chemotherapy, nerve-sparing radical hysterectomy significantly reduced the risk of bladder dysfunction (15.0% *versus* 32.9%, OR 0.31, 95%CI 0.14–0.68, *P* = 0.004). After propensity score matching, survival outcomes were similar with both types of adjuvant therapy (radiotherapy-based *versus* chemotherapy, *P*>0.05).

**Conclusion:**

Our study highlighted two distinct complication profiles of adjuvant therapy after radical hysterectomy for clinical stage IB-IIB cervical cancer, with radiotherapy increasing grade 3–4 adverse events and chemotherapy increasing bladder dysfunction. In this setting, nerve-sparing surgery may be useful if chemotherapy is being considered for adjuvant therapy.

## Introduction

Cervical cancer is the most common gynecologic malignancy in Japan, with an estimated 10,520 new cases of invasive cervical cancer diagnosed and 2,813 deaths from this disease in 2015 [1]. Nearly 75% of women with cervical cancer have stage I-II disease at diagnosis in Japan [2], and radical hysterectomy (RH) combined with pelvic lymphadenectomy is the most common treatment approach for stage I-II cervical cancer in Japan. In the United States, concurrent chemo-radiation is preferentially chosen as the primary treatment of patients with stage IIB cervical cancer. Contrary, in Japan women with stage IIB cervical cancer often undergo primary radical hysterectomy utilizing adjuvant therapy [3].

RH for early cervical cancer was first proposed by Wertheim in the 1890s and the surgical technique was modified by Meigs in the 1940s [4]. Over time, the complications and operative mortality of RH have been reduced by various improvements to surgical techniques and perioperative management. Recent reports suggest relatively low mortality and morbidity rates when type III RH is performed for early cervical cancer, including intraoperative complications in 1–3% of patients, grade 3–4 postoperative complications in 5–17%, but postoperative bladder dysfunction can be up to 30% [5–9].

The National Comprehensive Cancer Network (NCCN) and the Japan Society of Gynecologic Oncology (JSGO) recommend adjuvant therapy after primary surgical treatment for women with high-risk and intermediate-risk early cervical cancer [10, 11]. Concurrent chemo-radiotherapy (CCRT) is the most common type of adjuvant therapy (58%) for high-risk patients with cervical cancer in Japan followed by adjuvant chemotherapy alone (20%), and radiotherapy alone (9%) [3]. In contrast, systematic chemotherapy is the most common modality for adjuvant therapy for intermediate-risk patients (33%) [2].

This variability of adjuvant therapy in Japan is mainly due to concern about the toxicity of radiotherapy. Many studies have shown that performing radiotherapy after RH for early cervical cancer is associated with an increased risk of complications compared to surgery alone or definitive radiotherapy alone [12, 13].

Adjuvant chemotherapy has certain advantages compared to radiotherapy, particularly with respect to avoiding complications such as cystitis, proctitis, fibrosis, and fistula. This is important in Japan, because Japanese women with cervical cancer are generally thin and a lean body habitus is associated with increased radiation toxicity [14],[15]. However, there is little evidence from national statistics regarding the incidence and characteristics of complications related to each type of adjuvant therapy after surgical treatment of early cervical cancer.

Accordingly, the objective of this study was to examine the pattern of perioperative complications in relation to the type of adjuvant therapy after type III RH for clinical stage IB-IIB cervical cancer in Japan.

## Materials and methods

### Study design and patient eligibility

A nationwide retrospective study was conducted at 87 member institutions of the Japanese Gynecologic Oncology Group (JGOG-1070s) [16]. After Institutional Review Board approval (Tokai ethical committee:12R233) was obtained, the institutional cervical cancer database was utilized to examine patients with cervical cancer at each participating institution. In brief, we used the Japanese Gynecologic Oncology Group (JGOG) data, a nationwide study composed by the Committee on Treatment Guidelines for Cervical Cancer in Japan. All data were fully anonymized before access.

Eligible women had clinical stage IB-IIB cervical cancer and underwent type III RH with pelvic and/or para-aortic between January 1, 2008 and December 31, 2009. The data acquisition period at participating institutions was set from March 1 to August 31, 2012. This study captured consecutive cases of cervical cancer (maximum 10 cases) per in each institution during the study period to reduce selection-bias between institutions and reflects on the reality in clinical practice in Japan. Patients were excluded from this study if their lymph node status was unknown, if the cancer stage was unknown, or if the tumor was stage IA or stages III-IV. The Strengthening the Reporting of Observational Studies in Epidemiology (STROBE) guidelines for retrospective observational studies were followed [17].

### Clinical information

The following information was obtained from the medical records of eligible patients: *(i)* demographic data including the age and body mass index (BMI) at diagnosis, *(ii)* pathological data including the histological subtype, International Federation of Gynecology and Obstetrics (FIGO) cancer stage, tumor size, nodal involvement, and length of the resected vaginal cuff, *(iii)* treatment information including neoadjuvant therapy, surgical procedures (radical hysterectomy, bilateral salpingo-oophorectomy, para-aortic lymphadenectomy, number of resected lymph nodes, nerve-sparing technique, estimated blood loss, and operating time), and adjuvant therapy (radiotherapy and chemotherapy), *(iv)* perioperative complications from surgery to the final follow-up selected on the basis of previous studies (ureteral injury, bladder injury, bowel injury, surgical site fistula, urination difficulty, urinary incontinence, lymphocele, lymphedema, lymphorrhea, lower extremity cellulitis, thromboembolism, constipation, diarrhea, intestinal bleeding, and hematuria) [18], and *(V)* survival outcomes including disease-free survival (DFS) and cause-specific survival (CSS).

### Study definitions

Age and BMI were dichotomized according to previous studies [19]. Histologic subtypes were classed as either squamous or non-squamous. The resected vaginal cuff length was dichotomized at the median value. Complications were graded according to the National Cancer Institute’s Common Terminology Criteria for Adverse Events (CTC-AE) version 4.0 [20]. Intraoperative and postoperative complications were defined as adverse events of grade 3 or higher, which occurred the period of a patient's surgical procedure and the period between after surgery and last follow-up. Postoperative bladder dysfunction was defined as difficulty with urination or urinary incontinence requiring prolonged self-catheterization.

RH was either type III or type C hysterectomy, characterized by wide extirpation of parametrial tissue and separation of the posterior leaf of the vesico-uterine ligament [21]. Nerve-sparing surgery was defined as preservation of the vesical branches arising from the inferior hypogastric plexus while transecting the uterine branches of this plexus [22]. DFS was defined as the interval between surgery and first recurrence or death from cervical cancer, while CSS was the interval between surgery and death from cervical cancer. Patients without these survival events at final follow-up were censored.

### Statistical analysis

The primary outcome of this study was the frequency of perioperative adverse events (grade ≥3) associated with each postoperative treatment modality. Secondary outcomes were the frequency of adverse events for each clinical stage and the interaction of factors contributing to bladder dysfunction. In addition, survival was assessed based on adjuvant therapy types.

Continuous variables were expressed as the mean ± standard deviation (SD) or as the median with interquartile range (IQR). The significance of differences between continuous variables was assessed with the Kruskal-Wallis *H* test or one-way ANOVA as appropriate. Categorical and ordinal variables were expressed as the number (%), and the significance of differences was examined by the chi-square test.

Binary logistic regression models were used to identify independent risk factors for grade 3–4 perioperative complications or bladder dysfunction. Variables found to be significant on univariate analysis were entered into the initial model. The extent of significance was expressed as the adjusted odds ratio (OR) and its 95% confidence interval (CI).

Survival analysis was performed to examine the influence of adjuvant therapy on DFS and CSS after propensity score matching was performed to adjust for differences of background factors between the groups [23]. The propensity score was determined by multivariate logistic regression analysis, and an automated algorithm was used for one-to-one propensity score matching between the chemotherapy and radiotherapy-based groups. The optimal caliper width for estimating differences was calculated as one fifth of the standardized difference for the logit of the propensity score, resulting in a propensity score difference cut-off value of 0.03 in this study [24]. Standardized difference of ≤0.10 represented a good balance between the two groups after the matching.

The Kaplan-Meier method was utilized to construct survival curves, and the significance of differences between the curves was assessed by the log-rank test. Multivariate analysis with Cox proportional hazards regression models was used to identify independent prognostic factors for DFS and CSS. All the covariates found to be significant by univariate analysis were entered in the final model, and the extent of significance was expressed as the adjusted hazard ratio (HR) and its 95%CI.

The variance inflation factor (VIF) among the covariates in multivariate analysis was determined, and a VIF ≥2.0 was defined as indicating multicollinearity. All statistical tests were based upon two-tailed hypotheses, and *P*<0.05 was considered to indicate statistical significance. The Statistical Package for the Social Sciences (IBM SPSS, version 25.0, Armonk, NY, USA) was used for all analyses.

## Results

### Patient characteristics

Among 738 women with clinical stage IB-IIB cervical cancer who underwent RH and pelvic lymphadenectomy, lymph node evaluation was done in 693 women. Among those 693 patients, 405 (58.4%) were in stage IB1, 95 (13.7%) were in stage IB2, 72 (10.4%) were in stage IIA, and 121 (17.5%) were in stage IIB. The majority of tumors were squamous carcinoma (*n* = 447, 64.5%). Nearly half of the women underwent nerve-sparing surgery (*n* = 333, 48.1%), while para-aortic lymphadenectomy was not performed frequently (*n* = 100, 14.4%). Adjuvant therapy was administered to 427 (61.6%) women. Among 156 (22.5%) women who received postoperative chemotherapy, paclitaxel was used most frequently (*n* = 105, 67.3%), followed by carboplatin (*n* = 69, 44.2%) and cisplatin (*n* = 56, 35.9%). Among 271 women received postoperative RT, the vast majority received whole pelvic irradiation (*n* = 262, 96.7%) with a median dose of 50 Gy (IQR 45–54).

### Perioperative complications

The characteristics of women in each clinical stage are shown in Table 1. The overall intraoperative complication rate was 3.3% (*n* = 23, 95%CI 2.0–4.6), with the most common complication being bladder injury (*n* = 12, 1.7%), followed by ureteral injury (*n* = 10, 1.4%). The overall frequency of grade 3–4 postoperative complications was 9.8% (*n* = 68, 95%CI 7.6–12.0), with bowel obstruction (*n* = 35, 5.1%) being the most common, followed by lymphocele (*n* = 15, 2.2%), diarrhea (*n* = 7, 1.0%), and urinary tract fistula (*n* = 7, 1.0%). There were no grade 5 adverse events or grade 3–4 bladder dysfunction. The median time until recovery of urinary function was 14 days (IQR 10–22), and 86 patients (12.6%) needed to perform self-catheterization.

**Table 1:** Patients demographics per cancer stage.

Characteristic	All	Stage IB1	Stage IB2	Stage IIA	Stage IIB	*P*-value
Number	*N* = 693	*n* = 405	*n* = 95	*n* = 72	*n* = 121	
Age (years)	46 (38–57)	45 (38–57)	40 (34–48)	55 (43–61)	52 (42–60)	**<0.001**
BMI (kg/m^2^)	21.7 (19.6–23.9)	21.6 (19.6–23.6)	20.6 (19.4–24.6)	21.6 (19.6–24.2)	22.1 (19.9–24.3)	0.48
Tumor size (cm)	3.0 (2.0–4.1)	2.4 (1.5–3.0)	4.5 (4.1–5.2)	3.0 (2.5–4.2)	4.2 (3.2–5.0)	**<0.001**
Histologic subtype						0.63
SCC	447 (64.5%)	263 (64.9%)	57 (60.0%)	50 (69.4%)	77 (63.6%)	
Adenocarcinoma	163 (23.5%)	100 (24.7%)	22 (23.2%)	16 (22.2%)	25 (20.7%)	
Adenosquamous	42 (6.1%)	23 (5.7%)	7 (7.4%)	3 (4.2%)	9 (7.4%)	
Others	41 (5.9%)	19 (4.7%)	9 (9.5%)	3 (4.2%)	10 (8.3%)	
Neoadjuvant therapy						**<0.001**
No	624 (90.0%)	398 (98.3%)	79 (83.2%)	65 (90.3%)	82 (67.8%)	
Yes	69 (10.0%)	7 (1.7%)	16 (16.8%)	7 (9.7%)	39 (32.2%)	
Nerve sparing surgery						**0.001**
Not performed	360 (51.9%)	185 (45.7%)	52 (54.7%)	46 (63.9%)	77 (63.6%)	
Performed	333 (48.1%)	220 (54.3%)	43 (45.3%)	26 (36.1%)	44 (36.4%)	
Estimated blood loss (mL)	850 (550–1393)	800 (507–1275)	825 (502–1401)	938 (740–1354)	1117 (672–1690)	**<0.001**
Operative time (min)	320 (260–380)	310 (250–372)	332 (266–411)	278 (248–327)	345 (298–405)	**<0.001**
PAN dissection						**<0.001**
Not performed	593 (85.6%)	362 (89.4%)	77 (81.1%)	65 (90.3%)	89 (73.6%)	
Performed	100 (14.4%)	43 (10.6%)	18 (18.9%)	7 (9.7%)	32 (26.4%)	
Number of resected PLN/PAN	31 (22–43)	30 (22–40)	35 (25–53)	30 (23–45)	31 (22–43)	0.13
≤10	24 (3.5%)	10 (2.5%)	5 (5.3%)	3 (4.2%)	6 (5.0%)	
11–20	111 (16.0%)	74 (18.3%)	11 (11.6%)	11 (15.3%)	15 (12.4%)	
21–30	207 (29.9%)	123 (30.4%)	23 (24.2%)	23 (31.9%)	38 (31.4%)	
31–40	228 (32.9%)	134 (33.1%)	28 (29.5%)	26 (36.1%)	40 (33.1%)	
>40	123 (17.7%)	64 (15.8%)	28 (29.5%)	9 (12.5%)	22 (18.2%)	
Nodal involvement						**<0.001**
No	510 (73.6%)	368 (81.0%)	56 (58.9%)	53 (73.6%)	73 (60.3%)	
Yes	183 (26.4%)	77 (19.0%)	39 (41.1%)	19 (26.4%)	48 (39.7%)	
Length of vaginal cuff (cm)	2.5 (2.0–3.0)	2.5 (2.0–3.0)	2.9 (2.0–3.5)	3.0 (2.0–3.2)	2.5 (2.0–3.0)	**0.01**
Adjuvant therapy						**<0.001**
None	266 (38.4%)	213 (52.6%)	19 (20.0%)	20 (27.8%)	14 (11.6%)	
Radiotherapy alone	95 (13.7%)	47 (11.6%)	16 (16.8%)	12 (16.7%)	20 (16.5%)	
Chemotherapy alone	156 (22.5%)	69 (17.0%)	29 (30.5%)	17 (23.6%)	41 (33.9%)	
Both RT and CT	176 (25.4%)	76 (18.8%)	31 (32.6%)	23 (31.9%)	46 (38.0%)	
Intraoperative complication*	23 (3.3%)	11 (2.7%)	4 (4.2%)	1 (1.4%)	7 (5.8%)	0.28
Ureteral injury	10 (1.4%)	2 (0.5%)	1 (1.4%)	1 (1.4%)	5 (4.1%)	0.16
Bladder injury	12 (1.7%)	7 (1.7%)	3 (3.2%)	0	2 (1.7%)	0.42
Bowel injury	2 (0.3%)	2 (0.5%)	0	0	0	0.71
Postoperative hospital stays (day)	21 (16–31)	19 (15–27)	22 (15–36)	23 (17–52)	29 (18–49)	**<0.001**
Recovery of urinary function (day)	14 (10–20)	14 (10–20)	13 (10–20)	14 (10–21)	16 (10–24)	0.47
Postoperative complication*	68 (9.8%)	31 (7.7%)	12 (12.6%)	10 (13.9%)	15 (12.4%)	0.15
Urinary tract fistula	7 (1.0%)	4 (1.0%)	2 (2.1%)	0	1 (0.8%)	0.59
Difficulty urinating	0	0	0	0	0	
Urinary incontinence	0	0	0	0	0	
Lymphocele	15 (2.2%)	8 (2.0%)	2 (2.1%)	3 (4.2%)	2 (1.7%)	0.66
Lymphoedema	2 (0.3%)	1 (0.2%)	1 (1.1%)	0	0	0.47
Lymphorrhea	1 (0.1%)	1 (0.2%)	0	0	0	0.87
Cellulitis for legs	0	0	0	0	0	
Thromboembolic disease	4 (0.6%)	1 (0.2%)	1 (1.1%)	1 (1.4%)	1 (0.8%)	0.55
Bowel obstruction / ileus	35 (5.1%)	15 (3.7%)	4 (4.2%)	6 (8.3%)	10 (8.3%)	0.17
Constipation	0	0	0	0	0	
Diarrhea	7 (1.0%)	4 (1.0%)	0	1 (1.4%)	2 (1.7%)	0.67
Intestinal bleeding	0	0	0	0	0	
Hematuria	0	0	0	0	0	

Values are presented as median (interquartile range) or number (% per column). Kruskal-Wallis *H* test or chi-square test for *P*-values. Significant *P*-values are emboldened.

*items may duplicate.

Abbreviations: BMI, body mass index; PAN, para-aortic lymph node; PLN, pelvic lymph node; RT, radiotherapy; and CT, chemotherapy.

### Factors related to perioperative complications

According to univariate analysis, the factors associated with grade 3–4 intraoperative complications were para-aortic lymphadenectomy, a larger number of resected lymph nodes, and a longer operating time (all *P*<0.05) (Table 2). Clinical stage was not associated with an increased risk of grade 3–4 complications (*P*>0.05). Women who received radiotherapy had the highest incidence of grade 3–4 postoperative complications (14.7% with radiotherapy alone, 16.5% with radiotherapy plus chemotherapy, 7.1% with chemotherapy alone, and 5.3% with no adjuvant therapy; *P*<0.001). Adjuvant radiotherapy significantly increased the incidence of urinary tract fistula, lymphocele, and ileus (all *P*<0.05) (S1 Table). Multivariate analysis confirmed that radiotherapy was an independent risk factor for grade 3–4 postoperative complications (adjusted-ORs 3.19–3.26, *P*<0.05) (Table 3). On the other hand, chemotherapy was not associated with postoperative complications (*P* = 0.69).

**Table 2:** Grade 3–4 complications related to radical hysterectomy (univariate).

		Intraoperative		Postoperative	
Characteristic	No.	OR (95%CI)	*P*-value	OR (95%CI)	*P*-value
Age (years)					
< 60	557	1		1	
≥ 60	136	0.86 (0.29–2.57)	0.78	1.18 (0.64–2.16)	0.60
BMI (kg/m^2^)*					
< 25	565	1		1	
≥ 25	118	1.43 (0.52–3.95)	0.49	0.83 (0.41–1.67)	0.59
Tumor size (cm)					
< 4.0	449	1		1	
≥ 4.0	193	1.98 (0.84–4.65)	0.12	1.61 (0.94–2.75)	0.08
Unknown	51	n.a	0.99	1.18 (0.44–3.14)	0.75
Histology					
Squamous	447	1		1	
Non-squamous	246	0.79 (0.32–1.95)	0.61	0.92 (0.54–1.56)	0.76
Neoadjuvant therapy					
No	624	1		1	
Yes	69	0.86 (0.20–3.74)	0.84	0.86 (0.36–2.08)	0.74
Clinical stage					
IB1	405	1		1	
IB2	95	1.57 (0.49–5.06)	0.45	1.74 (0.86–3.54)	0.12
IIA	72	0.50 (0.06–3.97)	0.52	1.95 (0.91–4.17)	0.09
IIB	121	2.20 (0.83–5.80)	0.11	1.71 (0.89–3.28)	0.11
Nerve sparing surgery					
Not performed	360	1		1	
Performed	333	0.46 (0.19–1.14)	0.09	0.79 (0.47–1.30)	0.43
PAN dissection					
Not performed	593	1		1	
Performed	100	3.35 (1.38–8.13)	**0.007**	1.31 (0.67–2.54)	0.43
Number of resected PLN/PAN	693	1.02 (1.00–1.04)	**0.02**	0.99 (0.98–1.01)	0.80
Operative time (min)**	686	1.007 (1.00–1.01)	**<0.001**	1.003 (1.00–1.01)	**0.04**
Length of vaginal cuff (cm)					
< 2.5	242	1		1	
≥ 2.5	316	1.69 (0.63–4.51)	0.30	0.84 (0.48–1.47)	0.54
Unknown	135	1.20 (0.33–4.33)	0.78	0.89 (0.44–1.79)	0.73
Adjuvant therapy					
None	266			1	
Radiotherapy alone	95			3.11 (1.42–6.80)	**0.004**
Chemotherapy alone	156			1.37 (0.60–3.09)	0.45
Both RT and CT	176			3.55 (1.82–6.94)	**<0.001**

Binary logistic regression models for univariate analysis. Significant *P*-values are emboldened.

*10 cases are missing.

**7 cases are missing. There was multicollinearity between EBL and operative time, and EBL was excluded from the model.

Abbreviations: OR, odds ratio; CI, confidence interval; BMI, body mass index; PAN, para-aortic lymph node; PLN, pelvic lymph node; RT, radiotherapy; CT, chemotherapy; and n.a, not available.

**Table 3:** Surgical morbidities related to radical hysterectomy (multivariate).

		G3-4 intraoperative complication		G3-4 postoperative complication		Intraoperative urologic complication		Postoperative bladder dysfunction	
Characteristic	No.	OR (95%CI)	*P*-value	OR (95%CI)	*P*-value	OR (95%CI)	*P*-value	OR (95%CI)	*P*-value
Tumor size (cm)									
< 4.0	449							1	
≥ 4.0	193							1.17 (0.71–1.91)	0.54
Unknown	51							1.98 (0.92–4.29)	0.08
Nerve sparing surgery									
Not performed	360							1	
Performed	333							0.57 (0.37–0.90)	**0.02**
PAN dissection									
Not performed	593	1				1			
Performed	100	2.04 (0.74–5.63)	0.17			1.83 (0.62–5.37)	0.27		
Number of resected PLN/PAN	693	1.01 (0.99–1.03)	0.43			1.008 (0.99–1.03)	0.50	1.02 (1.00–1.03)	**0.03**
Operative time (min)*	686	1.006 (1.00–1.01)	**0.003**	1.003 (1.00–1.01)	**0.04**	1.007 (1.00–1.01)	**0.001**		
Length of vaginal cuff (cm)									
< 2.5	242							1	
≥ 2.5	316							1.42 (0.84–2.41)	0.19
Unknown	135							1.58 (0.85–2.94)	0.15
Adjuvant therapy									
None	266			1				1	
Radiotherapy alone	95			3.19 (1.46–6.99)	**0.004**			1.73 (0.87–3.42)	0.12
Chemotherapy only	156			1.19 (0.52–2.73)	0.69			2.06 (1.16–3.67)	**0.01**
Both RT and CT	176			3.26 (1.66–6.41)	**0.001**			1.19 (0.64–2.20)	0.59

Binary logistic regression models for multivariate analysis. Only significant variables in univariate analysis were entered in the final models. Significant *P*-values are emboldened.

*7 cases are missing.

Abbreviation: OR, odds ratio; CI, confidence interval; BMI, body mass index; PAN, para-aortic lymph node; PLN, pelvic lymph node; RT, radiotherapy; and CT, chemotherapy.

### Factors related to bladder dysfunction

Univariate analysis revealed that nerve-sparing surgery was associated with a significantly lower risk of postoperative bladder dysfunction compared to non nerve-sparing surgery (OR 0.64, 95%CI 0.42–0.97, *P* = 0.04) (Table 4). In addition, a larger tumor size, para-aortic lymphadenectomy, and a larger number of resected lymph nodes were associated with an increased risk of intraoperative urologic complications, including bladder and ureteral injury (all *P*<0.05). According to multivariate analysis, nerve-sparing surgery (adjusted-OR 0.57, 95%CI 0.37–0.90, *P* = 0.02), the number of resected lymph nodes (adjusted-OR 1.02, 95%CI 1.01–1.03, *P* = 0.03), and postoperative chemotherapy (adjusted-OR 2.06, 95%CI 1.16–3.67, *P* = 0.01) were independent determinants of postoperative bladder dysfunction.

**Table 4:** Urologic complication related to radical hysterectomy (univariate).

		Intraoperative		Postoperative bladder dysfunction	
Characteristic	No.	OR (95%CI)	*P*-value	OR (95%CI)	*P*-value
Age (years)					
< 60	557	1		1	
≥ 60	136	0.68 (0.20–2.33)	0.53	0.94 (0.56–1.60)	0.83
BMI (kg/m^2^) *					
< 25	565	1		1	
≥ 25	118	1.62 (0.58–4.55)	0.36	0.64 (0.34–1.18)	0.15
Tumor size (cm)					
< 4.0	481	1		1	
≥ 4.0	161	1.78 (0.74–4.30)	**0.20**	1.65 (1.05–2.59)	**0.03**
Unknown	51	na	0.99	1.64 (0.78–3.46)	0.19
Histology					
Squamous	447	1		1	
Non-squamous	246	0.91 (0.36–2.28)	0.83	0.81 (0.53–1.24)	0.34
Neoadjuvant therapy					
No	624	1		1	
Yes	69	0.95 (0.22–4.17)	0.95	0.70 (0.32–1.52)	0.37
Clinical stage					
IB1	405	1		1	
IB2	95	1.93 (0.58–6.42)	0.28	1.52 (0.84–2.74)	0.16
IIA	72	0.62 (0.08–4.97)	0.65	1.17 (0.58–2.37)	0.66
IIB	121	2.70 (0.99–7.41)	0.05	1.53 (0.89–2.61)	0.15
Nerve sparing surgery					
Not performed	360	1		1	
Performed	333	0.42 (0.16–1.10)	0.08	0.64 (0.42–0.97)	**0.04**
PAN dissection					
Not performed	593	1		1	
Performed	100	3.11 81.22–7.92)	**0.02**	1.97 (1.18–3.29)	**0.01**
Number of resected PLN/PAN	693	1.02 (1.00–1.04)	**0.03**	1.02 (1.01–1.04)	**<0.001**
Operative time (min)**	686	1.007 (1.00–1.01)	**<0.001**	1.00 (0.99–1.00)	0.97
Length of vaginal cuff (cm)					
< 2.5	242	1		1	
≥ 2.5	316	1.42 (0.52–3.89)	0.50	1.71 (1.04–2.83)	**0.04**
Unknown	135	1.20 (0.33–4.33)	0.78	1.98 (1.10–3.58)	**0.02**
Adjuvant therapy					
None	266			1	
Radiotherapy alone	95			1.66 (0.85–3.21)	0.14
Chemotherapy alone	156			2.54 (1.49–4.33)	**0.001**
Both RT and CT	176			1.29 (0.72–2.30)	0.39

Binary logistic regression models for univariate analysis. Significant *P*-values are emboldened.

*10 cases are missing.

**7 cases are missing. There was multicollinearity between EBL and operative time, and EBL was excluded from the model.

Abbreviations: OR, odds ratio; CI, confidence interval; BMI, body mass index; PAN, para-aortic lymph node; PLN, pelvic lymph node; RT, radiotherapy; and CT, chemotherapy.

The interaction between nerve-sparing surgery and chemotherapy was examined (Table 5). According to multivariate analysis, nerve-sparing RH was associated with a significantly lower risk of bladder dysfunction among women receiving postoperative chemotherapy (15.0% *versus* 32.9%, adjusted-OR 0.31, 95%CI 0.14–0.68, *P* = 0.004).

**Table 5:** Incidence of postoperative bladder dysfunction based on nerve-sparing surgery and chemotherapy use.

Characteristic	Number (%)	Bladder dysfunction (%)	OR (95%CI)	*P*-value
Combination patterns				
Chemo (+) / nerve-sparing (-)	76 (11.0%)	25 (32.9%)	1	
Chemo (+) / nerve-sparing (+)	80 (11.5%)	12 (15.0%)	0.31 (0.14–0.68)	**0.004**
Chemo (-) / nerve-sparing (-)	284 (41.0%)	40 (14.1%)	0.37 (0.20–0.67)	**0.001**
Chemo (-) / nerve-sparing (+)	253 (36.5%)	29 (11.5%)	0.28 (0.15–0.52)	**<0.001**
Number of resected nodes	693 (100%)	n.a	1.02 (1.01–1.04)	**<0.001**

The incidence of bladder dysfunction is shown based on combination patterns of nerve-sparing surgery during radical hysterectomy and postoperative chemotherapy use. A binary logistic regression model for multivariate analysis: all the listed covariates were entered in the final model. Significant *P*-values are emboldened. Abbreviation: OR, odds ratio; chemo, adjuvant chemotherapy; and n.a, not available.

### Survival outcomes

Survival analysis was performed in the 693 women with cervical cancer. The median follow-up time of censored cases in the whole cohort was 40.0 months (IQR 36.4–44.8) and 69 women died of cervical cancer during the follow-up period. According to multivariate analysis (S2 Table), higher clinical stage, non-squamous histology, non-nerve sparing RH, and lymph node metastasis were independent predictors for decreased survival (all adjusted *P*<0.05). On the other hand, adjuvant therapy was not an independent predictor of an increased risk of cause-specific mortality. However, tumor characteristics were significantly different between the women who received postoperative therapy and those who only underwent surgery. Therefore, we subsequently performed stratified analysis.

Survival outcomes were examined in the 427 women who received postoperative therapy. The median follow-up time of censored cases was 40.2 months (IQR 37.9–45.9) for the whole cohort and 62 women died of cervical cancer during the follow-up period. After propensity score matching (S3 Table), demographic factors were similar between the chemotherapy and radiotherapy-based groups (all, *SD*<0.1). The chemotherapy group and the radiotherapy-based group showed similar survival rates (5-year DFS rates, 67.6% *versus* 63.3%, *P* = 0.67; and 5-year CSS rates, 79.4% *versus* 77.0%, *P* = 0.92 S1A and S1B Fig). On multivariate analysis (S4 Table), higher clinical stage, non-squamous histology, and lymph node metastasis were independent predictors for decreased survival (all adjusted *P*<0.05). In addition, the influence of nerve-sparing surgery and chemotherapy on survival was examined (S5 Table). According to multivariate analysis, nerve-sparing RH without postoperative chemotherapy was associated with significantly better survival (5-year CSS rates 95.7%, adjusted-OR 0.24, 95%CI 0.10–0.55, *P* = 0.001).

## Discussion

Our study identified two distinct complication profiles related to adjuvant therapy after RH for clinical stage IB-IIB cervical cancer. In brief, we found that radiotherapy was associated with more grade 3–4 adverse events, whereas chemotherapy was associated with a higher risk of bladder dysfunction. Additionally, our results suggested that nerve-sparing surgery reduced bladder dysfunction in women receiving adjuvant chemotherapy.

Randomized clinical trials have demonstrated a survival benefit of adjuvant radiotherapy for high-risk and intermediate-risk early cervical cancer (GOG-92 and GOG-109) [14, 25]. A recent JGOG survey showed that chemotherapy was the most common adjuvant therapy for intermediate-risk early cervical cancer, and nearly one third (33.1%) of JGOG-affiliated institutions treat this disease with adjuvant chemotherapy alone [3]. Other JGOG studies performed in patients with high-risk and intermediate-risk early cervical cancer have demonstrated similar survival with postoperative adjuvant chemotherapy and postoperative radiotherapy [26, 27].

While postoperative adjuvant therapy may improve survival in women with early cervical cancer, it is associated with an increased risk of severe adverse events after RH [18, 28]. In fact, various adverse events have been reported after type III RH [12–14, 29]. In particular, radiotherapy is known to be associated with an increased risk of various complications after RH [12, 18]. Grade 3–4 adverse events were reported in 6–18% of women with stage IB-IIA cervical cancer who received adjuvant radiotherapy following radical hysterectomy [14] [25]. The present study also demonstrated similar findings, since radiotherapy was associated with the highest postoperative complication rate of nearly 15% after RH with different types of adjuvant therapy, including chemotherapy. It has been reported that performing chemotherapy after RH may increase the risk of intestinal and urologic complications [5]. Postoperative urinary dysfunction after RH may be attributable to damage to the pelvic autonomic nerves, so nerve-sparing surgery was previously suggested to reduce the risk of urinary complications [22]. Our results support the concept that nerve-sparing RH may reduce postoperative bladder dysfunction related to chemotherapy-induced peripheral neuropathy [29, 30]. Conversely, women who received postoperative chemotherapy without nerve-sparing RH had a markedly higher incidence of postoperative bladder dysfunction (32.9%).

Since survival was similar with adjuvant radiotherapy or chemotherapy, treatment-related complications that can significantly affect the quality-of-life are an important consideration when choosing adjuvant therapy [26],[27]. A recent randomized clinical trial compared postoperative chemotherapy (paclitaxel/cisplatin) with CCRT for high-risk and intermediate-risk early cervical cancer, showing comparable survival in the two treatment arms but better quality-of-life with postoperative chemotherapy [31]. Because these results are favor for chemotherapy as the choice for adjuvant therapy, performing nerve-sparing RH could be a key to reducing chemotherapy-related bladder dysfunction. In Japan, the JGOG is currently conducting a phase III clinical trial that compares CCRT with chemotherapy after RH for high-risk clinical stage IB–IIB cervical cancer (JGOG-1060) [16], and the trial should provide useful information regarding the efficacy and toxicity of adjuvant chemotherapy in this population.

The strengths of the present study include one of the largest sample sizes reporting perioperative complication to date and sufficient events to perform multivariate analysis. Complication rates are similar to what reported in literature, but the statistics demonstrated in this nationwide large cohort is more valid and informative. In addition, this study was conducted at JGOG-affiliated institutions with accredited quality of care [32]. Limitations include a retrospective design and the possibility of unknown confounders influencing the results of analysis. For example, the reasons for selecting each type of adjuvant therapy were not included in the database. Another weakness is that this study lacks information regarding preoperative bladder function, and thus, the direct association between radical hysterectomy and postoperative bladder function remain unknown. In addition, this study did not evaluate the objective quality of RH or nerve-sparing surgery.

A clinical application of our findings may be in the area of postoperative treatment. This study clearly showed that adjuvant radiotherapy increased the risk of grade 3–4 postoperative complications after type III RH whereas chemotherapy did not, which can be useful information for selecting adjuvant therapy after RH. In addition, adjuvant chemotherapy was associated with an increased risk of bladder dysfunction, while nerve-sparing RH reduced this risk in patients receiving adjuvant chemotherapy. Thus, nerve-sparing surgery would preferably be performed when chemotherapy is to be given postoperatively. Further investigation will be required to confirm these results.

## Supporting information

S1 Fig Survival curves based on adjuvant treatment type. Kaplan-Meier method for survival curves: (A) cause-specific survival and (B) disease-free survival. Log-rank test for P-values. Abbreviations: RT, radiotherapy; and CT, chemotherapy.
(PDF)

S1 Table Incidence of postoperative G3-4 complications based on postoperative adjuvant therapy. Percent per column. Chi-square test for P-values. Significant P-values are emboldened. *items may duplicate. Abbreviations: RT, radiotherapy; CT, chemotherapy; and n.a. not available.
(PDF)

S2 Table Multivariate analysis in survival for women in cervical cancer after radical hysterectomy (n = 693). A Cox proportional hazard regression model for multivariate analysis. Significant covariates in the univariate analysis were initially entered the multivariate model and conditional backward was performed. Tumor size was not included in the models due to staging factor (VIF ≥2.0). *6 cases were missing original data. Significant P-values are emboldened. Abbreviations: HR, Hazard ratio; 95%CI, 95% confidence interval; 5-yr (%), 5-year survival; BMI, body mass index; SCC, squamous cell carcinoma; PAN, para-aortic lymph node; PLN, pelvic lymph node; RT, radiotherapy; CT, chemotherapy; and n.a, not available.
(PDF)

S3 Table Demographics for propensity score matching. Number (%) per column or median (interquartile range) is shown. Standardized differences are shown before and after propensity score matching. *6 cases were missing original data and 3 cases were missing after propensity score matching. Abbreviations: SD, standardized difference; and BMI, body mass index.
(PDF)

S4 Table Multivariate analysis for survival after propensity score matching. A Cox proportional hazard regression model for multivariate analysis. Significant covariates in the univariate analysis were initially entered the multivariate model. Significant P-values are emboldened. Abbreviations: HR, Hazard ratio; 95%CI, 95% confidence interval; 5-yr (%), 5-year survival; BMI, body mass index; and PAN, para-aortic lymph node.
(PDF)

S5 Table Multivariate analysis in survival for women in cervical cancer based on nerve-sparing surgery and chemotherapy use. Cox proportional hazard regression models for multivariable analysis is shown based on combination patterns of nerve-sparing surgery during radical hysterectomy and postoperative chemotherapy use. Significant covariates in the univariate analysis were initially entered the multivariate model. Significant P-values are emboldened. Abbreviations: 5-yr (%), cumulative risk of 5-year; HR, Hazard ratio; 95%CI, 95% confidence interval; SCC, squamous cell carcinoma; Chemo, adjuvant chemotherapy; and n.a. not available.
(PDF)
