# Supplementary material for: Profile of treatment-related complications in women with clinical stage IB-IIB cervical cancer: A nationwide cohort study in Japan
Source: PLoS One. 2019 Jan 7;14(1):e0210125. doi: 10.1371/journal.pone.0210125 (PMC6322763; doi:10.1371/journal.pone.0210125)

**Supplemental Figure S1. Survival curves based on adjuvant treatment type.**

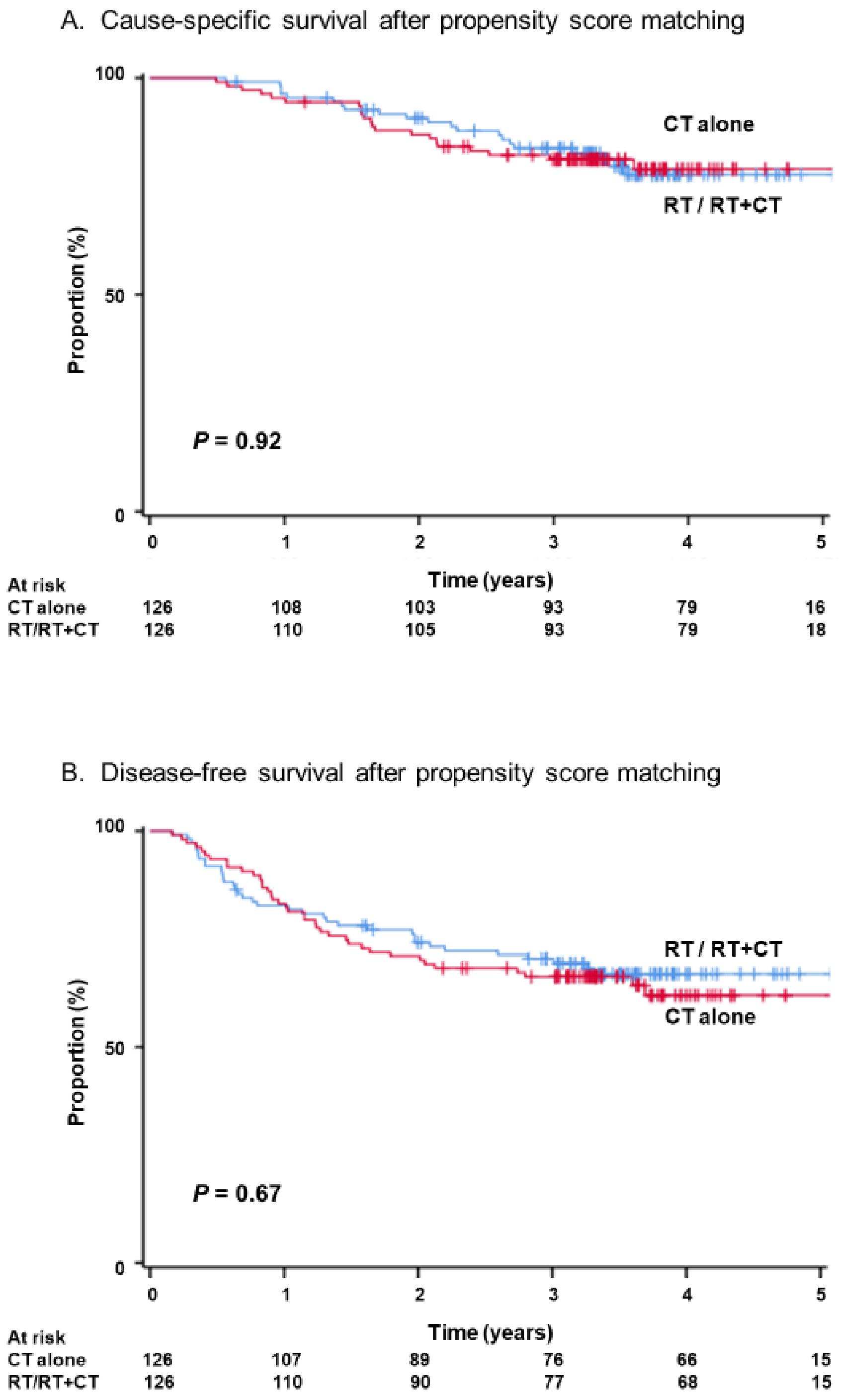

Supplement: S1 Fig — Kaplan-Meier method for survival curves: (A) cause-specific survival and (B) disease-free survival. Log-rank test for P-values. Abbreviations: RT, radiotherapy; and CT, chemotherapy. (PDF) [file pone.0210125.s001.pdf]
