# Supplementary material for: Profile of treatment-related complications in women with clinical stage IB-IIB cervical cancer: A nationwide cohort study in Japan
Source: PLoS One. 2019 Jan 7;14(1):e0210125. doi: 10.1371/journal.pone.0210125 (PMC6322763; doi:10.1371/journal.pone.0210125)
Supplement: S1 Table — Percent per column. Chi-square test for P-values. Significant P-values are emboldened. *items may duplicate. Abbreviations: RT, radiotherapy; CT, chemotherapy; and n.a. not available. (PDF) [file pone.0210125.s002.pdf]

Supplemental Table S1. Incidence of postoperative G3-4 complications based on postoperative adjuvant therapy.

Characteristic	All	None	RT alone	CT alone	Both RT / CT	*P*-value
Number	*N*=693	*n*=266	*n*=95	*n*=156	*n*=176	
Postoperative complication*	68 (9.8%)	(5.3%)	(14.7%)	(7.1%)	(16.5%)	**0.001**
Urinary tract fistula	7 (1.0%)	1 (0.4%)	0	1 (0.6%)	5 (2.8%)	**0.02**
Difficulty urinating	0	0	0	0	0	n.a
Urinary incontinence	0	0	0	0	0	n.a
Lymphocele	15 (2.2%)	4 (1.5%)	4 (4.2%)	0	7 (4.0%)	**0.04**
Lymphoedema	2 (0.3%)	1 (0.4%)	0	0	1 (0.6%)	0.74
Lymphorrhea	1 (0.1%)	0	1 (1.1%)	0	0	0.10
Cellulitis for legs	0	0	0	0	0	n.a
Thromboembolic disease	4 (0.6%)	0	1 (1.1%)	3 (1.9%)	0	**0.049**
Bowel obstruction / ileus	35 (5.1%)	6 (2.3%)	7 (7.4%)	5 (3.2%)	17 (9.7%)	**0.003**
Constipation	0	0	0	0	0	n.a
Diarrhea	7 (1.0%)	0	2 (2.1%)	0	5 (2.8%)	0.01
Intestinal bleeding	0	0	0	0	0	n.a
Hematuria	0	0	0	0	0	n.a
