# Supplementary material for: Profile of treatment-related complications in women with clinical stage IB-IIB cervical cancer: A nationwide cohort study in Japan
Source: PLoS One. 2019 Jan 7;14(1):e0210125. doi: 10.1371/journal.pone.0210125 (PMC6322763; doi:10.1371/journal.pone.0210125)
Supplement: S2 Table — A Cox proportional hazard regression model for multivariate analysis. Significant covariates in the univariate analysis were initially entered the multivariate model and conditional backward was performed. Tumor size was not included in the models due to staging factor (VIF ≥2.0). *6 cases were missing original data. Significant P-values are emboldened. Abbreviations: HR, Hazard ratio; 95%CI, 95% confidence interval; 5-yr (%), 5-year survival; BMI, body mass index; SCC, squamous cell carcinoma; PAN, para-aortic lymph node; PLN, pelvic lymph node; RT, radiotherapy; CT, chemotherapy; and n.a, not available. (PDF) [file pone.0210125.s003.pdf]

**Supplemental Table S2. Multivariate analysis in survival for women in cervical cancer after radical hysterectomy (n=693).**

| | Disease-free survival | | | | | Cause-specific survival | | | | |
|---|---|---|---|---|---|---|---|---|---|---|
| | 5-yr | Univariate | | Multivariate | | 5-yr | Univariate | | Multivariate | |
| Characteristics | (%) | HR (95%CI) | *P*-value | HR (95%CI) | *P*-value | (%) | HR (95%CI) | *P*-value | HR (95%CI) | *P*-value |
| Age (years) | | | | | | | | | | |
| < 60 | 75.1 | 1 | | 1 | | 86.1 | 1 | | | |
| ≥ 60 | 84.1 | 0.57 (0.34-0.96) | **0.04** | 0.56 (0.33-0.95) | **0.03** | 91.4 | 0.56 (0.27-1.18) | 0.13 | | |
| BMI (kg/m$^2$)* | | | | | | | | | | |
| < 25 | 77.5 | 1 | | | | 88.0 | 1 | | | |
| ≥ 25 | 77.8 | 1.00 (0.63-1.59) | 0.99 | | | 85.3 | 1.06 (0.57-1.98) | 0.86 | | |
| Tumor size (cm) | | | | | | | | | | |
| < 4.0 | 81.8 | 1 | | | | 92.8 | 1 | | | |
| ≥ 4.0 | 61.7 | 2.46 (1.74-3.49) | **<0.001** | | | 71.6 | 4.39 (2.66-7.26) | **<0.001** | | |
| Unknown | 92.6 | 0.42 (0.13-1.35) | 0.15 | | | 97.5 | 0.37 (0.05-2.77) | 0.34 | | |
| Histologic subtype | | | | | | | | | | |
| SCC | 82.0 | 1 | | 1 | | 90.2 | 1 | | 1 | |
| Adenocarcinoma | 70.6 | 1.62 (1.09-2.42) | **0.02** | 1.93 (1.28-2.90) | **0.002** | 83.4 | 1.67 (0.95-2.91) | 0.07 | 2.13 (1.20-3.77) | **0.01** |
| Adenosquamous | 67.1 | 1.87 (0.99-3.54) | 0.055 | 1.78 (0.93-3.38) | 0.08 | 85.7 | 1.28 (0.45-3.63) | 0.64 | 1.31 (0.46-3.73) | 0.61 |
| Others | 56.6 | 3.07 (1.75-5.39) | **<0.001** | 2.62 (1.49-4.60) | **0.001** | 67.2 | 4.70 (2.37-9.32) | **<0.001** | 4.27 (2.14-8.53) | **<0.001** |
| Neoadjuvant therapy | | | | | | | | | | |
| No | 77.4 | 1 | | | | 87.5 | 1 | | | |
| Yes | 70.8 | 1.33 (0.79-2.24) | 0.29 | | | 83.9 | 1.40 (0.69-2.83) | 0.35 | | |
| Clinical stage | | | | | | | | | | |
| IB1 | 85.3 | 1 | | 1 | | 94.2 | 1 | | 1 | |
| IB2 | 62.5 | 3.15 (2.01-4.94) | **<0.001** | 2.32 (1.46-3.68) | **<0.001** | 74.3 | 4.83 (2.59-8.98) | **<0.001** | 3.12 (1.66-5.88) | **<0.001** |
| IIA | 65.5 | 2.30 (1.34-3.96) | **0.003** | 1.84 (1.06-3.22) | **0.03** | 82.8 | 2.30 (1.01-5.25) | **0.048** | 1.43 (0.61-3.35) | 0.41 |
| IIB | 67.6 | 2.48 (1.59-3.89) | **<0.001** | 1.91 (1.20-3.06) | **0.007** | 76.8 | 3.55 (1.88-6.71) | **<0.001** | 1.96 (1.01-3.81) | **0.047** |
| Nerve sparing surgery | | | | | | | | | | |
| Not performed | 71.1 | 1 | | 1 | | 80.9 | 1 | | 1 | |
| Performed | 82.6 | 0.53 (0.37-0.76) | **<0.001** | 0.59 (0.41-0.85) | **0.004** | 93.6 | 0.33 (0.19-0.58) | **<0.001** | 0.36 (0.20-0.62) | **0.001** |
| Estimated blood loss (mL) | n.a | 1.00 (0.99-1.00) | 0.06 | | | n.a | 1.00 (0.99-1.01) | 0.08 | | |
| Operative time (min) | n.a | 1.00 (0.99-1.00) | 0.45 | | | n.a | 1.00 (0.99-1.01) | 0.81 | | |
| PAN dissection | | | | | | | | | | |
| Not performed | 77.9 | 1 | | | | 87.8 | 1 | | | |
| Performed | 70.1 | 1.41 (0.90-2.19) | 0.13 | | | 82.6 | 1.55 (0.84-2.84) | 0.16 | | |
| Number of resected PLN/PAN | n.a | 1.01 (0.99-1.02) | 0.09 | | | n.a | 1.01 (0.99-1.02) | 0.08 | | |
| Nodal involvement | | | | | | | | | | |
| No | 84.8 | 1 | | 1 | | 93.0 | 1 | | 1 | |
| Yes | 55.6 | 3.55 (2.51-5.02) | **<0.001** | 3.01 (2.10-4.31) | **<0.001** | 71.3 | 5.48 (3.33-9.04) | **<0.001** | 2.81 (2.84-8.12) | **<0.001** |

| | | | | | | |
|---|---|---|---|---|---|---|
| Length of vaginal cuff (cm) | | | | | | |
| < 2.5 | 76.4 | 1 | | 88.5 | 1 | |
| ≥ 2.5 | 77.5 | 0.98 (0.66-1.45) | 0.91 | 85.8 | 1.18 (0.68-2.05) | 0.56 |
| Unknown | 75.6 | 1.04 (0.64-1.68) | 0.88 | 88.0 | 1.05 (0.53-2.11) | 0.89 |
| Adjuvant therapy | | | | | | |
| None | 88.8 | 1 | | 95.8 | 1 | |
| Radiotherapy alone | 82.1 | 1.50 (0.75-3.02) | 0.25 | 93.1 | 2.04 (0.65-6.44) | 0.22 |
| Chemotherapy alone | 66.2 | 3.45 (2.08-5.71) | **<0.001** | 77.8 | 6.21 (2.69-14.4) | **<0.001** |
| Both RT and CT | 66.2 | 3.47 (2.12-5.69) | **<0.001** | 80.6 | 6.43 (2.82-14.6) | **<0.001** |
| Intraoperative complication* | | | | | | |
| No | 77.3 | 1 | | 87.3 | 1 | |
| Yes | 61.6 | 1.93 (0.94-3.94) | 0.07 | 80.8 | 1.80 (0.66-4.95) | 0.25 |
| Postoperative hospital stays (day) | n.a | 1.01 (1.00-1.02) | **<0.001** | n.a | 1.01 (1.00-1.02) | **0.02** |
| Recovery of urinary function (day) | n.a | 1.00 (0.99-1.00) | 0.79 | n.a | 0.99 (0.99-1.00) | 0.49 |
| Postoperative complication* | | | | | | |
| No | 77.2 | 1 | | 87.5 | 1 | |
| Yes | 76.5 | 1.10 (0.76-1.59) | 0.61 | 86.0 | 1.28 (0.78-2.09) | 0.33 |
