# Supplementary material for: Profile of treatment-related complications in women with clinical stage IB-IIB cervical cancer: A nationwide cohort study in Japan
Source: PLoS One. 2019 Jan 7;14(1):e0210125. doi: 10.1371/journal.pone.0210125 (PMC6322763; doi:10.1371/journal.pone.0210125)
Supplement: S3 Table — Number (%) per column or median (interquartile range) is shown. Standardized differences are shown before and after propensity score matching. *6 cases were missing original data and 3 cases were missing after propensity score matching. Abbreviations: SD, standardized difference; and BMI, body mass index. (PDF) [file pone.0210125.s004.pdf]

Supplemental Table S3. Demographics for propensity score matching.

	Before propensity score matching				After propensity score matching			
		Chemotherapy	Radiotherapy-based	*SD*		Chemotherapy	Radiotherapy-based	*SD*
Characteristics	No.	*n*=156	*n*=271		No.	*n*=126	*n*=126	
Age (years)				0.113				0.082
< 60	341	129 (82.7%)	212 (78.2%)		201	105 (83.3%)	101 (80.2%)	
≥ 60	86	27 (17.3%)	59 (21.8%)		47	21 (16.7%)	25 (19.8%)	
BMI (kg/m^2)*				0.001				0.088
< 25	349	126 (82.9%)	223 (82.9%)		204	102 (82.3%)	106 (85.5%)	
≥ 25	72	26 (17.1%)	46 (17.1%)		41	22 (17.7%)	18 (14.5%)	
Histology				0.276				0.049
Squamous	274	69 (44.2%)	84 (31.0%)		149	80 (63.5%)	77 (61.1%)	
Non-squamous	153	87 (55.8%)	187 (69.0%)		99	46 (36.5%)	49 (38.9%)	
Neoadjuvant therapy				0.146				0.049
Not performed	377	133 (85.3%)	244 (90.0%)		223	112 (88.9%)	110 (87.3%)	
Performed	50	23 (14.7%)	27 (10.0%)		25	14 (11.1%)	16 (12.7%)	
Nerve sparing surgery				0.193				0.048
Not performed	234	76 (48.7%)	158 (58.3%)		135	68 (54.0%)	71 (56.3%)	
Performed	193	80 (51.3%)	113 (41.7%)		113	58 (46.0%)	55 (43.7%)	
PAN dissection				0.227				0.085
Not performed	362	124 (79.5%)	238 (87.8%)		211	107 (84.9%)	103 (81.7%)	
Performed	65	32 (20.5%)	33 (12.2%)		37	19 (15.1%)	23 (18.3%)	
Length of vaginal cuff (cm)								0.099
< 2.5	128	33 (21.2%)	95 (35.1%)		67	33 (26.2%)	39 (31.0%)	
≥ 2.5	209	84 (53.8%)	125 (46.1%)		130	62 (49.2%)	59 (46.8%)	
unknown	90	39 (25.0%)	51 (18.8%)		51	31 (24.6%)	28 (22.2%)	
Clinical stage				0.033				0.084
IB1	192	69 (44.2%)	123 (45.4%)		124	58 (46.0%)	61 (48.4%)	
IB2	76	29 (18.6%)	47 (17.3%)		42	22 (17.5%)	26 (20.6%)	
IIA	52	17 (10.9%)	35 (12.9%)		27	15 (11.9%)	11 (8.7%)	
IIB	107	41 (26.3%)	66 (24.4%)		55	31 (24.6%)	28 (22.2%)	
Nodal involvement				0.092				0.033
No	245	94 (60.3%)	151 (55.7%)		149	78 (61.9%)	76 (60.3%)	
Yes	182	62 (36.7%)	120 (44.3%)		99	48 (38.1%)	50 (39.7%)	
