# Supplementary material for: Profile of treatment-related complications in women with clinical stage IB-IIB cervical cancer: A nationwide cohort study in Japan
Source: PLoS One. 2019 Jan 7;14(1):e0210125. doi: 10.1371/journal.pone.0210125 (PMC6322763; doi:10.1371/journal.pone.0210125)
Supplement: S4 Table — A Cox proportional hazard regression model for multivariate analysis. Significant covariates in the univariate analysis were initially entered the multivariate model. Significant P-values are emboldened. Abbreviations: HR, Hazard ratio; 95%CI, 95% confidence interval; 5-yr (%), 5-year survival; BMI, body mass index; and PAN, para-aortic lymph node. (PDF) [file pone.0210125.s005.pdf]

Supplemental Table S4. Multivariate analysis for survival after propensity score matching.

	Disease-free survival					Cause-specific survival				
	5-yr	Univariate		Multivariate		5-yr	Univariate		Multivariate	
Characteristics	(%)	HR (95%CI)	*P*-value	HR (95%CI)	*P*-value	(%)	HR (95%CI)	*P*-value	HR (95%CI)	*P*-value
Age (years)										
< 60	62.4	1				77.1	1			
≥ 60	72.7	0.63 (0.31-1.26)	0.19			83.0	0.61 (0.24-1.56)	0.30		
BMI (kg/m^2)										
< 25	67.2	1				80.9	1			
≥ 25	55.2	1.49 (0.83-2.68)	0.18			76.7	1.39 (0.64-3.00)	0.41		
Histology										
Squamous	74.0	1		1		84.5	1		1	
Non-squamous	49.4	2.10 (1.32-3.33)	**0.002**	2.51 (1.55-4.07)	**<0.001**	68.7	2.14 (1.16-3.94)	**0.01**	2.59 (1.36-4.91)	**0.004**
Neoadjuvant therapy										
Not performed	65.4	1				78.3	1			
Performed	54.1	1.38 (0.71-2.69)	0.35			77.4	1.10 (0.43-2.80)	0.84		
Nerve sparing surgery										
Not performed	67.8	1				68.7	1			
Performed	71.7	0.58 (0.36-1.03)	0.10			79.4	0.29 (0.14-1.60)	0.15		
PAN dissection										
Not performed	65.9	1				79.1	1			
Performed	55.9	1.36 (0.78-2.39)	0.28			74.2	1.35 (0.65-2.82)	0.43		
Length of vaginal cuff (cm)										
< 2.5	56.4	1				75.6	1			
≥ 2.5	64.5	0.81 (0.45-1.37)	0.43			78.3	0.86 (0.43-1.76)	0.69		
unknown	71.7	0.63 (0.32-1.22)	0.17			80.6	0.78 (0.33-1.86)	0.58		
Clinical stage										
IB1	76.5	1		1		87.0	1		1	
IB2	55.4	2.25 (1.23-4.60)	**0.01**	2.22 (1.22-4.06)	**0.01**	75.0	2.15 (0.96-4.80)	0.06	2.16 (1.10-4.85)	**0.04**
IIA	40.7	2.21 (1.05-4.65)	**0.04**	1.51 (0.71-3.24)	0.29	66.8	1.94 (1.07-6.72)	**0.04**	1.79 (0.70-4.59)	0.22
IIB	46.8	2.01 (1.10-3.66)	**0.02**	1.99 (1.09-3.63)	**0.03**	71.6	1.86 (0.85-4.21)	0.12	1.92 (0.86-4.32)	0.11
Nodal involvement										
No	74.2	1		1		85.9	1		1	
Yes	49.8	2.35 (1.48-3.85)	**<0.001**	2.62 (1.62-4.25)	**<0.001**	66.9	3.14 (1.67-5.91)	**<0.001**	3.51 (1.83-6.73)	**<0.001**
Adjuvant therapy										
Chemotherapy only	67.6	1				79.4	1			
Radiotherapy-based	63.3	0.86 (0.56-1.31)	0.67			77.0	0.97 (0.53-1.77)	0.92		
