# Supplementary material for: Profile of treatment-related complications in women with clinical stage IB-IIB cervical cancer: A nationwide cohort study in Japan
Source: PLoS One. 2019 Jan 7;14(1):e0210125. doi: 10.1371/journal.pone.0210125 (PMC6322763; doi:10.1371/journal.pone.0210125)
Supplement: S5 Table — Cox proportional hazard regression models for multivariable analysis is shown based on combination patterns of nerve-sparing surgery during radical hysterectomy and postoperative chemotherapy use. Significant covariates in the univariate analysis were initially entered the multivariate model. Significant P-values are emboldened. Abbreviations: 5-yr (%), cumulative risk of 5-year; HR, Hazard ratio; 95%CI, 95% confidence interval; SCC, squamous cell carcinoma; Chemo, adjuvant chemotherapy; and n.a. not available. (PDF) [file pone.0210125.s006.pdf]

Supplemental TableS5. Multivariate analysis in survival for women in cervical cancer based on nerve-sparing surgery and chemotherapy use.

	Disease-free survival					Cause-specific survival				
	5-yr	Univariate		Multivariate		5-yr	Univariate		Multivariate	
Characteristic	(%)	HR (95%CI)	*P*-value	HR (95%CI)	*P*-value	(%)	HR (95%CI)	*P*-value	HR (95%CI)	*P*-value
Combination patterns										
Chemo (+) / nerve-sparing (-)	60.9	1		1		74.0	1		1	
Chemo (+) / nerve-sparing (+)	71.3	0.65 (0.36-1.18)	0.16	0.69 (0.38-1.27)	0.23	86.7	0.40 (0.17-0.94)	**0.03**	0.41 (0.17-0.98)	**0.046**
Chemo (-) / nerve-sparing (-)	74.0	0.61 (0.38-0.97)	**0.04**	0.83 (0.51-1.34)	0.45	82.8	0.51 (0.28-0.91)	**0.02**	0.79 (0.42-1.47)	0.45
Chemo (-) / nerve-sparing (+)	86.2	0.28 (0.17-0.49)	**<0.001**	0.43 (0.25-0.75)	**0.003**	95.7	0.14 (0.06-0.32)	**<0.001**	0.24 (0.10-0.55)	**0.001**
Number of resected nodes	n.a	1.01 (0.99-1.02)	0.09			n.a	1.01 (0.99-1.02)	0.08		
Histologic subtype										
SCC	82.0	1		1		90.2	1		1	
Adenocarcinoma	70.6	1.62 (1.09-2.42)	**0.02**	1.81 (1.20-2.73)	**0.004**	83.4	1.67 (0.95-2.91)	0.07	2.03 (1.14-3.64)	**0.02**
Adenosquamous	67.1	1.87 (0.99-3.54)	0.055	1.88 (0.99-3.57)	0.056	85.7	1.28 (0.45-3.63)	0.64	1.35 (0.47-3.88)	0.58
Others	56.6	3.07 (1.75-5.39)	**<0.001**	2.62 (1.49-4.63)	**0.001**	67.2	4.70 (2.37-9.32)	**<0.001**	4.07 (2.01-8.24)	**<0.001**
Clinical stage										
IB1	85.3	1		1		94.2	1		1	
IB2	62.5	3.15 (2.01-4.94)	**<0.001**	2.22 (1.3903.53)	**0.001**	74.3	4.83 (2.59-8.98)	**<0.001**	2.97 (1.57-5.62)	**0.001**
IIA	65.5	2.30 (1.34-3.96)	**0.003**	1.73 (0.99-3.01)	0.054	82.8	2.30 (1.01-5.25)	**0.048**	1.39 (0.59-3.25)	0.45
IIB	67.6	2.48 (1.59-3.89)	**<0.001**	1.72 (1.08-2.74)	**0.02**	76.8	3.55 (1.88-6.71)	**<0.001**	1.91 (0.98-3.69)	0.06
Nodal involvement										
No	84.8	1		1		93.0	1		1	
Yes	55.6	3.55 (2.51-5.02)	**<0.001**	2.97 (2.07-4.26)	**<0.001**	71.3	5.48 (3.33-9.04)	**<0.001**	4.58 (2.71-7.77)	**<0.001**
